# Supplementary material for: Transfer of dysbiotic gut microbiota has beneficial effects on host liver metabolism
Source: Mol Syst Biol. 2017 Mar 16;13(3):921. doi: 10.15252/msb.20167356 (PMC5371731; doi:10.15252/msb.20167356)
Supplement: Supplementary file 1 — Appendix [file MSB-13-921-s001.pdf]

Table of Contents

- **Table S1 : Primer list**
- **Appendix Figures and legends**

Table S1. Primer sequences (5'-3') used in the study.

**Muc-2**	Forward	ATGCCCACCTCCTCAAAGAC
	Reverse	GTAGTTTCCGTTGGAACAGTGAA
**Claudin-2**	Forward	CCCAGGCCATGATGGTGA
	Reverse	TCATGCCCACCACAGAGATAAT
**Claudin-7**	Forward	CCTGGTGTTGGGCTTCTTAGC
	Reverse	CCCACAGCGTGTGCACTTC
**Jam-A**	Forward	CTGATCTTTGACCCCGTGAC
	Reverse	ACCAGACGCCAAAAATCAAG
**Occludin**	Forward	ATGTCCGGCCGATGCTCTC
	Reverse	TTTGGCTGCTCTTGGGTCTGTAT
**ZO-1**	Forward	ACCCGAAACTGATGCTGTGGATAG
	Reverse	AAATGGCCGGGCAGAACTTGTGTA
**FoxP3**	Forward	CCCATCCCCAGGAGTCTTG
	Reverse	ACCATGACTAGGGGCACTGTA
**IL-17a**	Forward	CTCCAGAAGGCCCTCAGACTAC
	Reverse	GGGTCTTCATTGCGGTGG
**IFN γ**	Forward	CGGCACAGTCATTGAAAGCC
	Reverse	TGTCACCATCCTTTTGCCAGT
**NF-kB1**	Forward	ATGGCAGACGATGATCCCTAC
	Reverse	TGTTGACAGTGGTATTTCTGGTG
**Reg-3γ**	Forward	CCATCTTCACGTAGCAGC
	Reverse	CAAGATGTCCTGAGGGC
**Reg-3β**	Forward	TGGGAATGGAGTAACAATG
	Reverse	GGCAACTTCACCTCACAT
**TNF-α**	Forward	CATCTTCTCAAAATTCGAGTGACAA
	Reverse	TGGGAGTAGACAAGGTACAACCC
**IL-1β**	Forward	TCGCTCAGGGTCACAAGAAA
	Reverse	CATCAGAGGCAAGGAGGAAAAC
**PAI-1**	Forward	ACAGCCTTTGTCATCTCAGCC
	Reverse	CCGAACCACAAAGAGAAAGGA
**iNOS**	Forward	CAGCTGGGCTGTACAAACCTT
	Reverse	CATTGGAAGTGAAGCGTTTCG
**IL-12p40**	Forward	GGAAGCACGGCAGCAGAATA
	Reverse	AACTTGAGGGAGAAGTAGGAATGG
**IL-6**	Forward	ACAAGTCGGAGGCTTAATTACACAT
	Reverse	TTGCCATTGCACAACTCTTTTC

Basal metabolism of donor and recipient mice

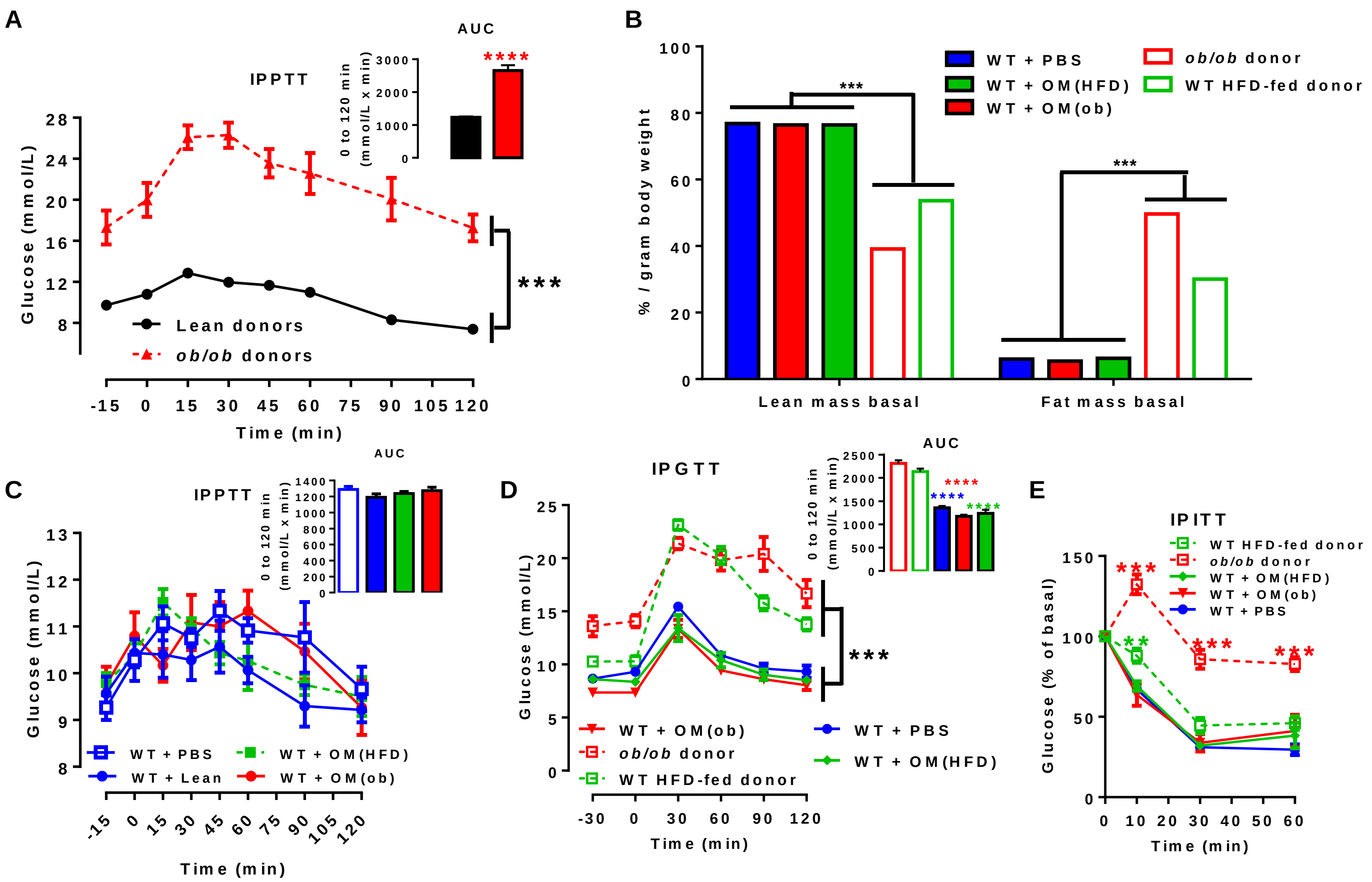

Appendix Figure S1

Donor gut (cecum) microbiota analysis

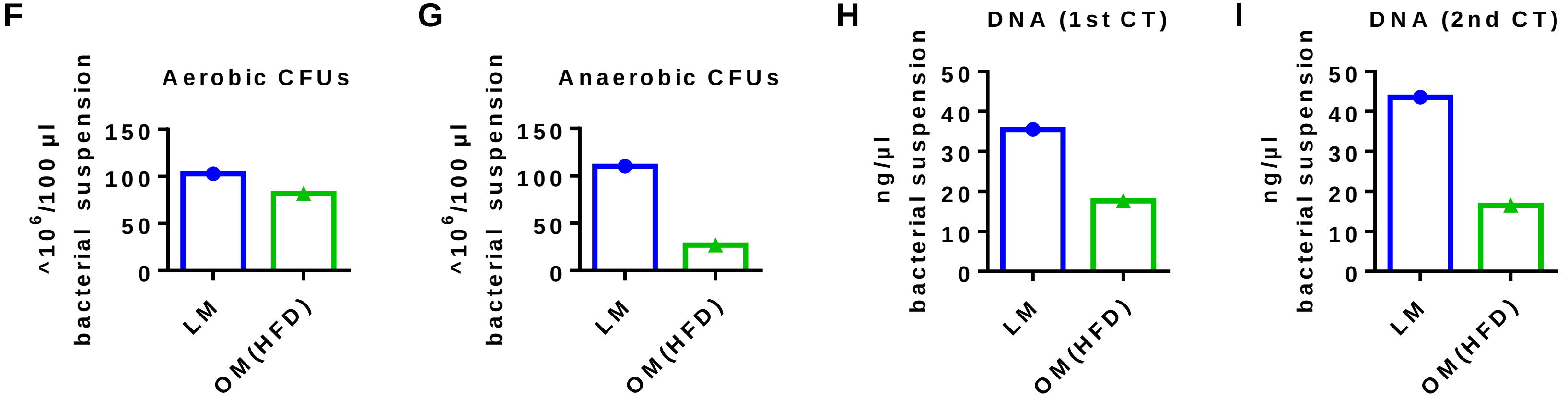

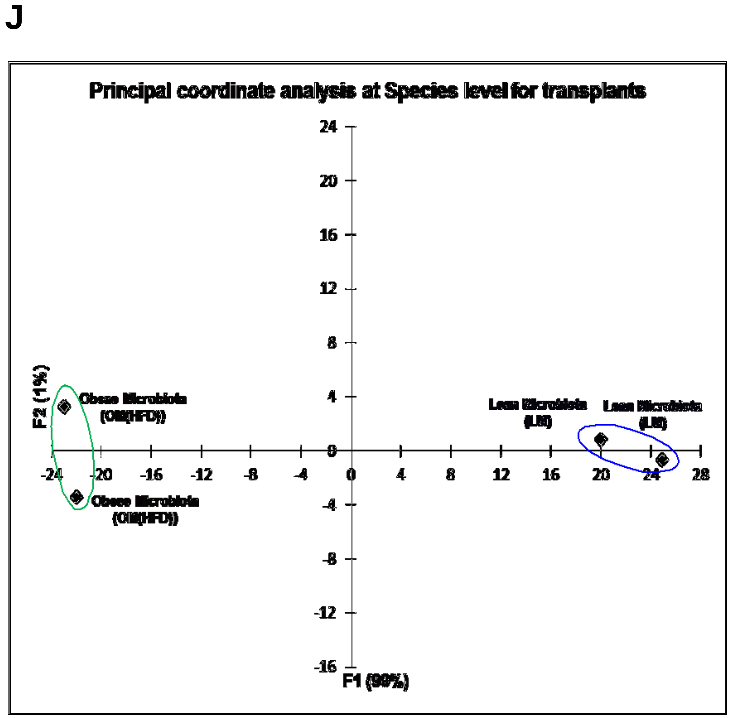

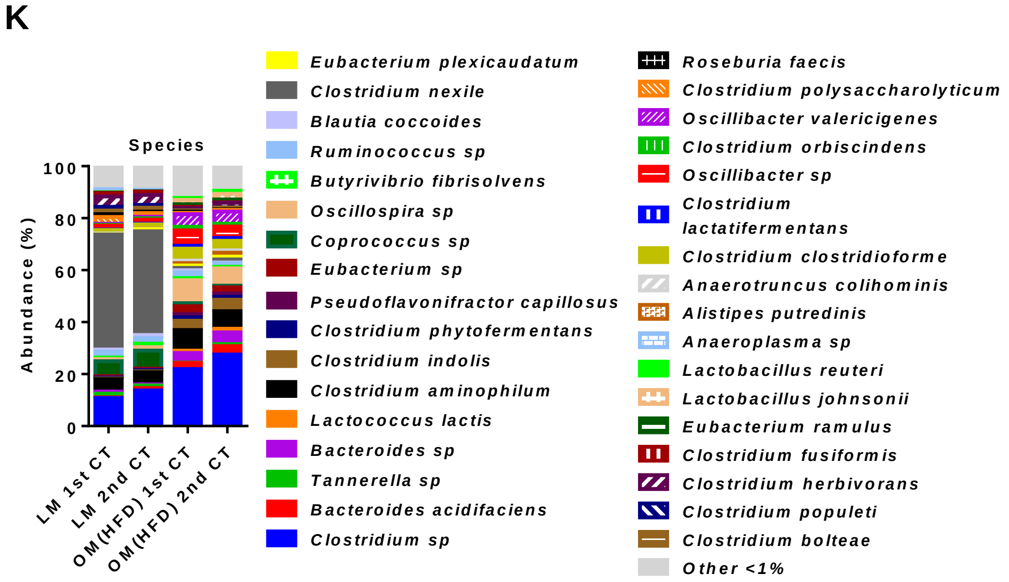

Appendix Figure S1

Appendix Figure S1. Basal metabolic features for both donor and recipient mice and characterization and comparison between eubiotic (lean) and dysbiotic (obese) gut microbiota used as transplants in *Protocol#1* and A) IPPTT for both lean and *ob/ob* donors and AUC as inset; B) lean and fat mass (%) for both donor and recipient mice; C) IPPTT for recipient mice and AUC as inset; D) IPGTT for both donor and recipient mice and AUC as inset; E) IPITT for both donor and recipient mice; enumeration on Petri dish cultivation of bacterial suspensions from both lean microbiota (LM) and HFD-induced dysbiotic microbiota (obese microbiota, OM(HFD)) in aerobic (F) and anaerobic condition (G). Spectrophotometry dosage of total DNA in bacterial suspension for the 1st cecal transfer (1st CT, H) and the second one (2nd CT, I); J) Principal coordinate analysis comparison between cecal transplants at the level of Species; K) pyrosequencing of *Species* shown as abundance (%) of total identified sequences per group (average of 3000 sequences per sample); Data as mean±SEM; **p<0,01; ***p<0,001; ****p<0,0001, 2-ANOVA with Sidak post test. A) n=20 for lean donors and 6 for *ob/ob* donors; B) n=6 per group except for donors: 4 *ob/ob* and 10 HFD-fed mice; C) n=6 per group; D) and E) as B).

Protocol #1 | All parameters on 6 hours fasted NC-fed mice

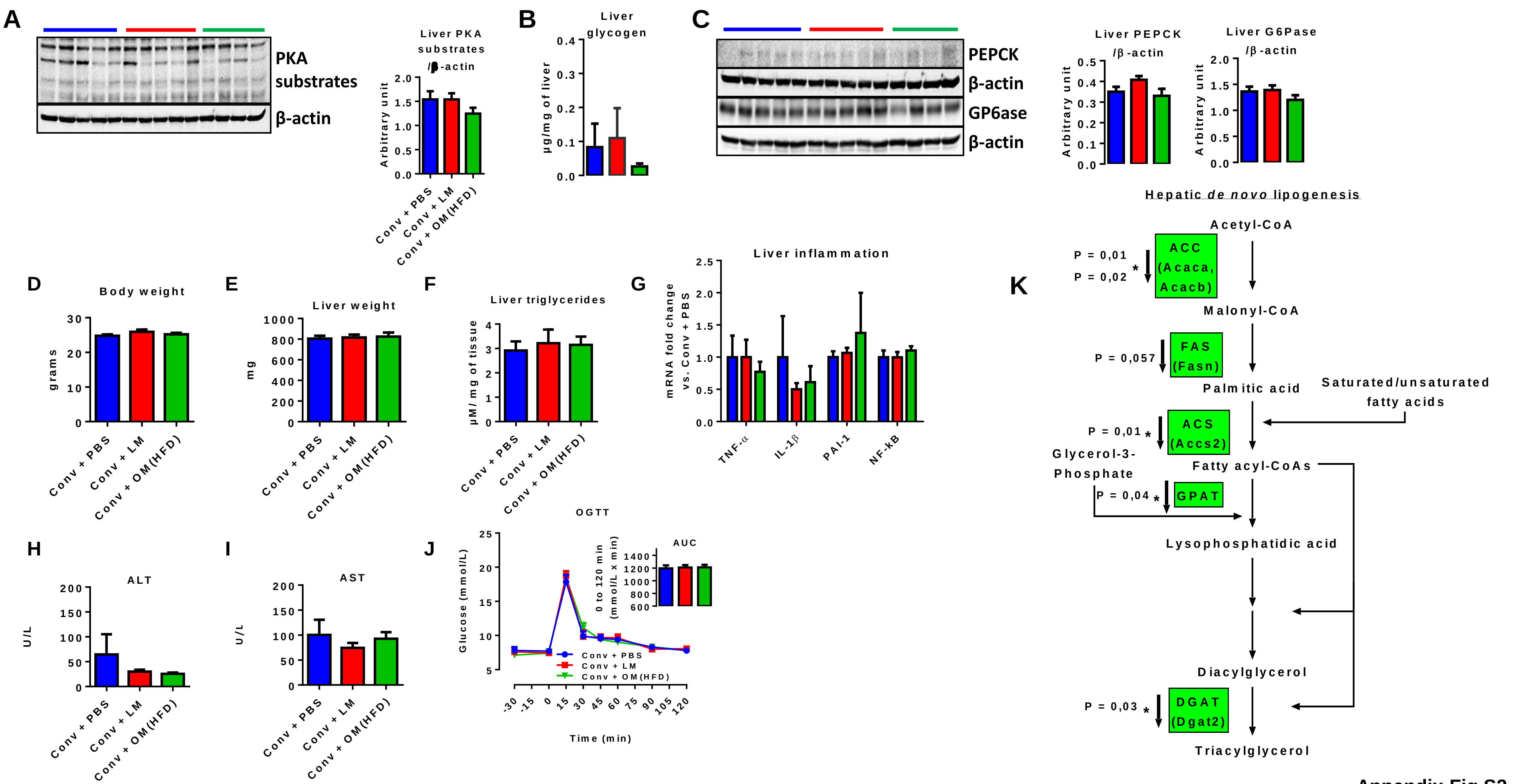

Appendix Fig.S2

Appendix Figure S2. Metabolic consequences during transfer of dysbiotic vs. eubiotic gut microbiota in NC-fed conventional mice.

A) liver extracts western-blot analysis for PKA substrates phosphorylation normalized on β-actin (load control) and related histograms; B) hepatic glycogen dosage; C) liver western-blot analyses for PEPCK and G6Pase normalized on β-actin (load control) and related histograms; D) body weight; liver E) weight and F) triglycerides; G) inflammatory genes; plasma levels for H) ALT and I) AST J) OGTT and AUC as inset; K) hepatic de novo lipogenesis pathway in antibiotic-free NC-fed conventional mice transferred with either the vehicle or cecal microbiota from C57Bl/6 lean or HFD-fed mice (Conv + PBS, Conv + OM(LM), Conv + OM(HFD), respectively). Data are shown as mean ± SEM; (n=5-6).

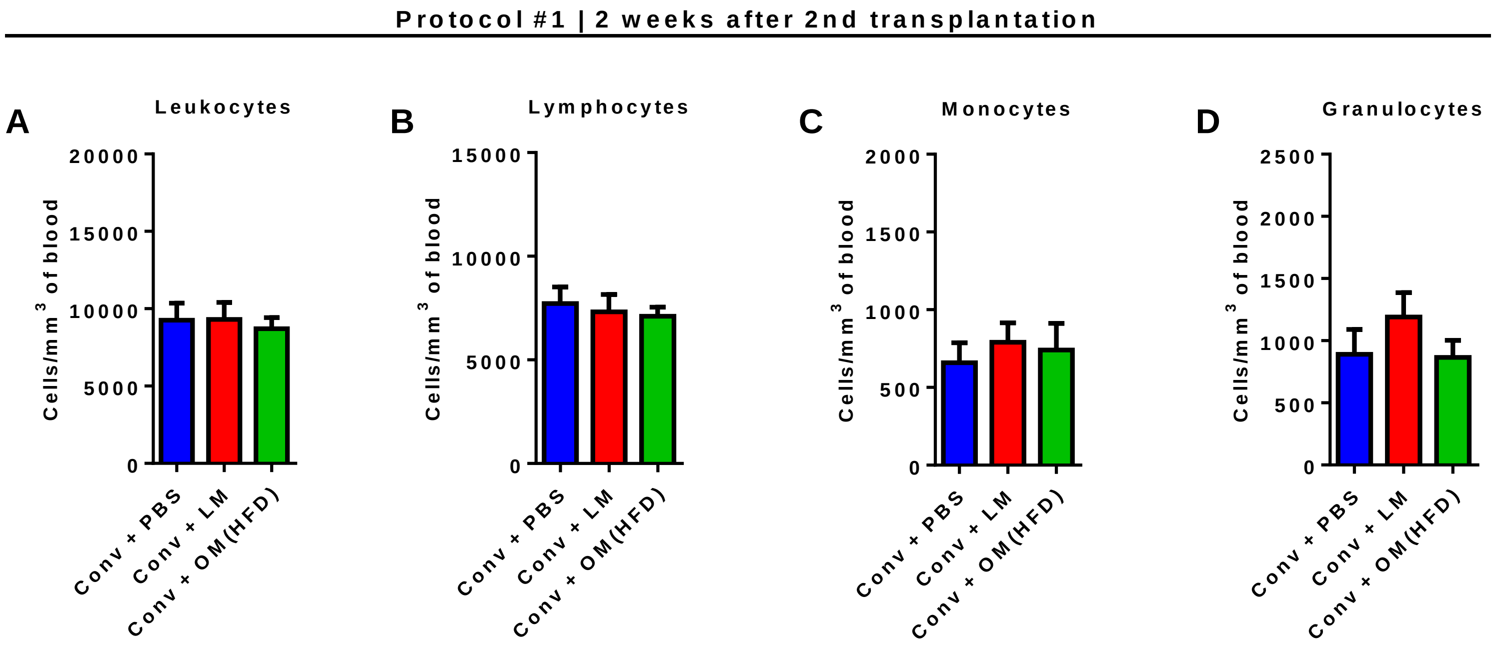

Appendix Figure S3. Systemic inflammatory tone analysis during transfer of dysbiotic vs. eubiotic gut microbiota in NC-fed conventional mice.

Plasma A) leukocytes; B) lymphocytes; C) monocytes and D) granulocytes enumeration 2 weeks after the 2nd transfer in antibiotic-free NC-fed conventional mice transferred with either the vehicle or cecal microbiota from NC-fed lean or HFD-fed obese mice (Conv + PBS, Conv + LM, Conv + OM(HFD), respectively). Data are shown as mean ± SEM; (n=6).

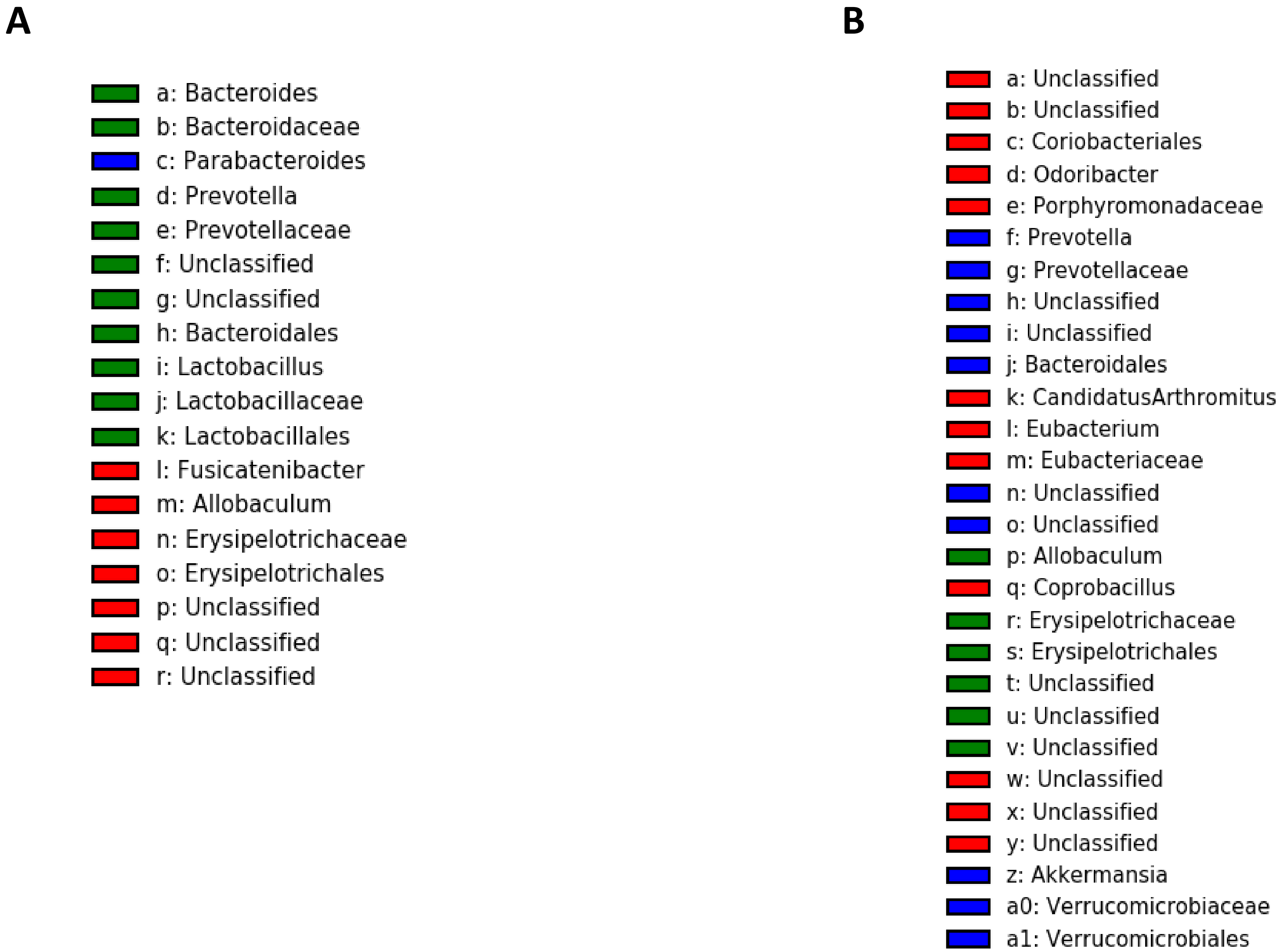

Appendix Figure S4. Full list for cladograms reported in Figure 4.
A) Lower panel for Fig.4A; B) Lower panel for Fig.4B.

Protocol #1

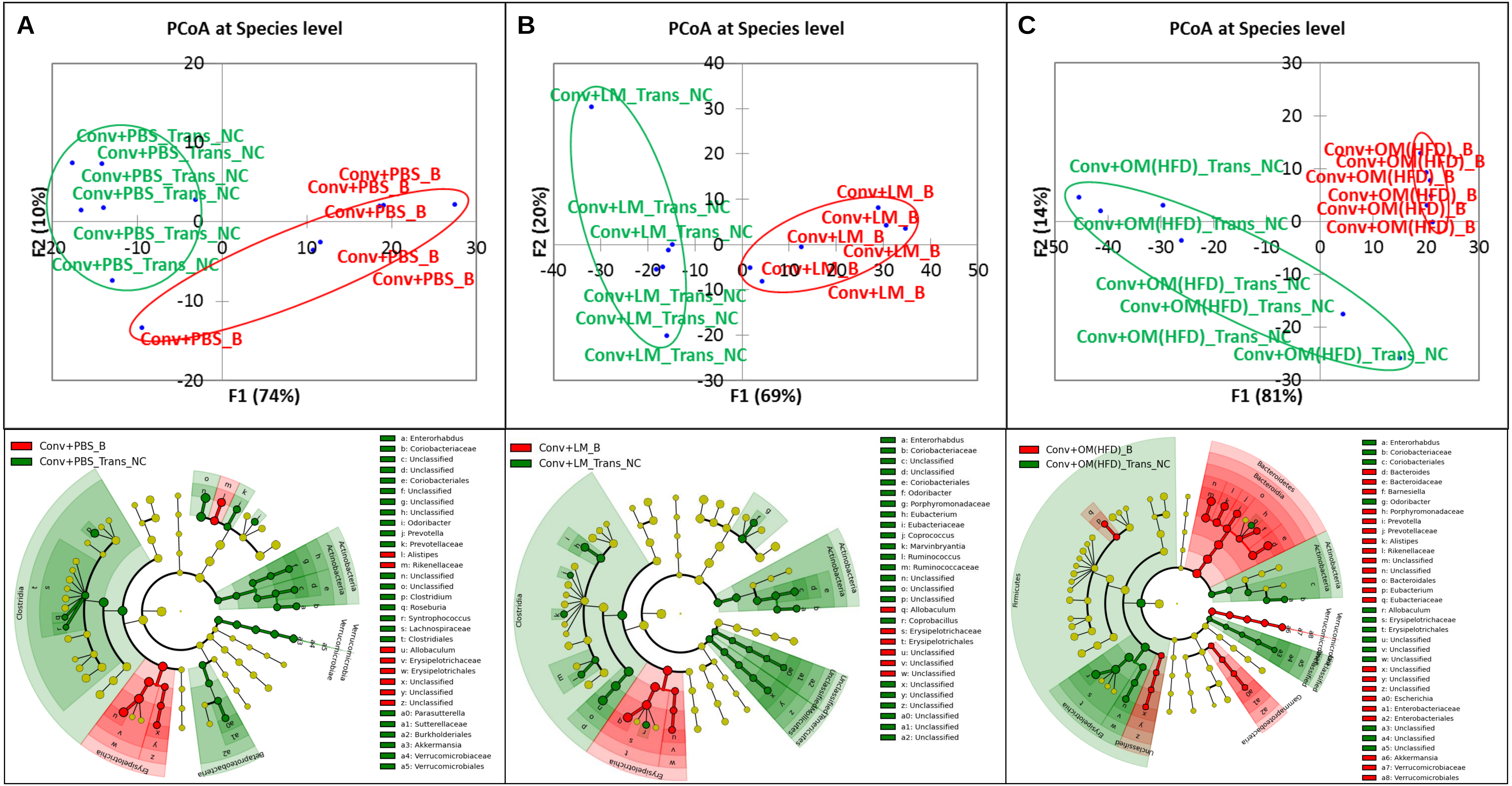

Appendix Figure S5

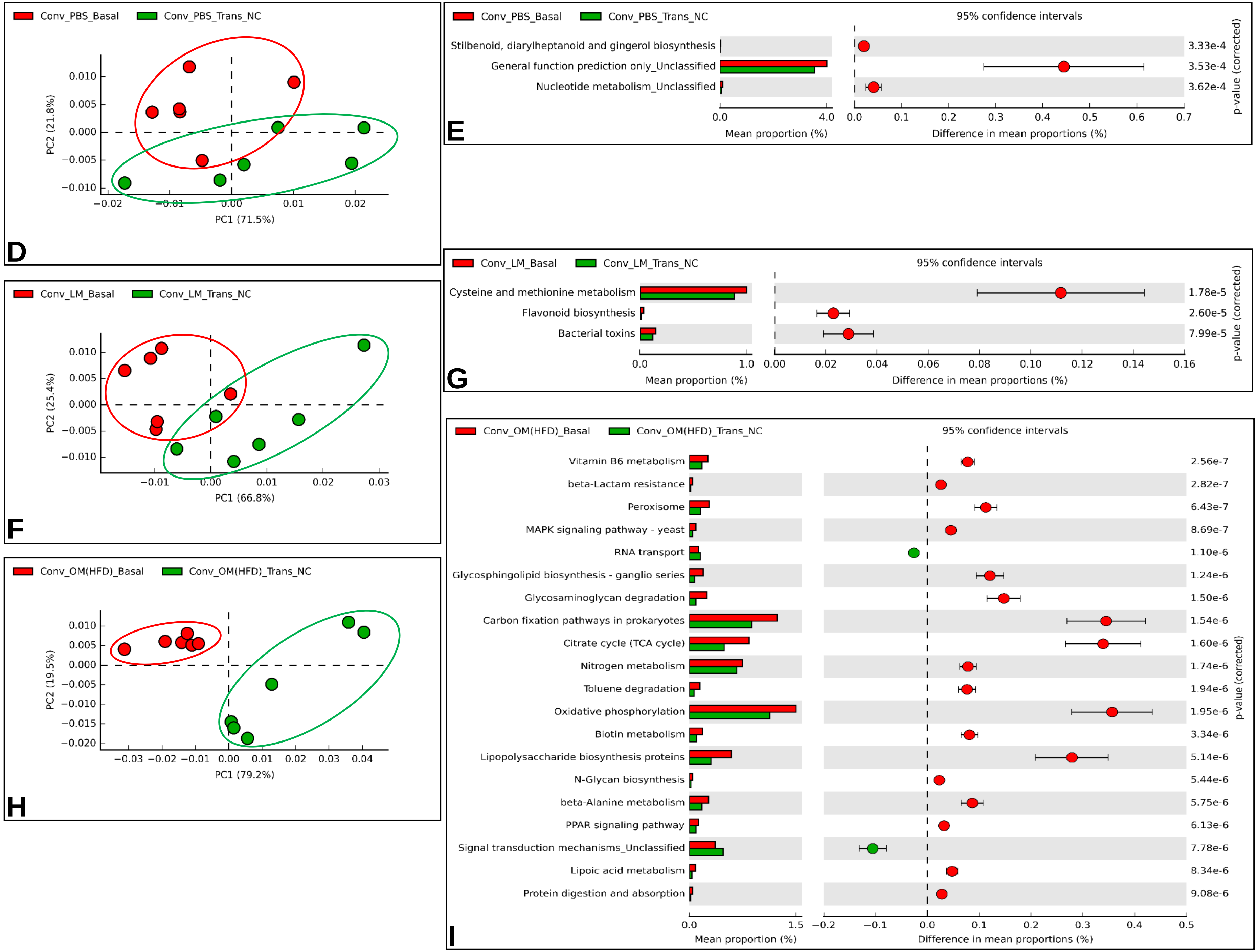

Appendix Figure S5

Appendix Figure S5. Transfer of dysbiotic vs. eubiotic gut microbiota in antibiotic-free NC-fed conventional mice differently affects gut microbiota and microbiome.

A) Principal coordinate analysis (PCoA) for gut microbiota profiles at baseline (Basal, B) and after transfer with the vehicle (PBS) on NC (Trans NC) (upper panel) and related cladograms showing bacterial taxa significantly enriched in each group (lower panel); B) PCoA for gut microbiota profiles at baseline (Basal, B) and after transfer with the lean microbiota (LM) on NC (Trans NC) (upper panel) and related cladogram (lower panel); C) PCoA for gut microbiota profiles at baseline (Basal, B) and after transfer with the HFD-microbiota (OM(HFD)) on NC (Trans NC) (upper panel) and related cladogram (lower panel); Principal Component Analysis showing PICRUSt-based gut microbiome study at baseline (Basal) and after transfer with the vehicle (PBS, D) or lean microbiota (LM, F) or HFD-microbiota (OM(HFD), H) on NC (Trans NC) and top modulated (based on the Two sided Welch's t-test) microbial pathways in a pair-wise comparison (E, G, I) in antibiotic-free NC-fed conventional mice inoculated with either the vehicle or cecal microbiota from C57Bl/6 lean or HFD-fed mice (Conv + PBS, Conv + LM, Conv +OM(HFD), respectively); (n=5-6).

Protocol #2

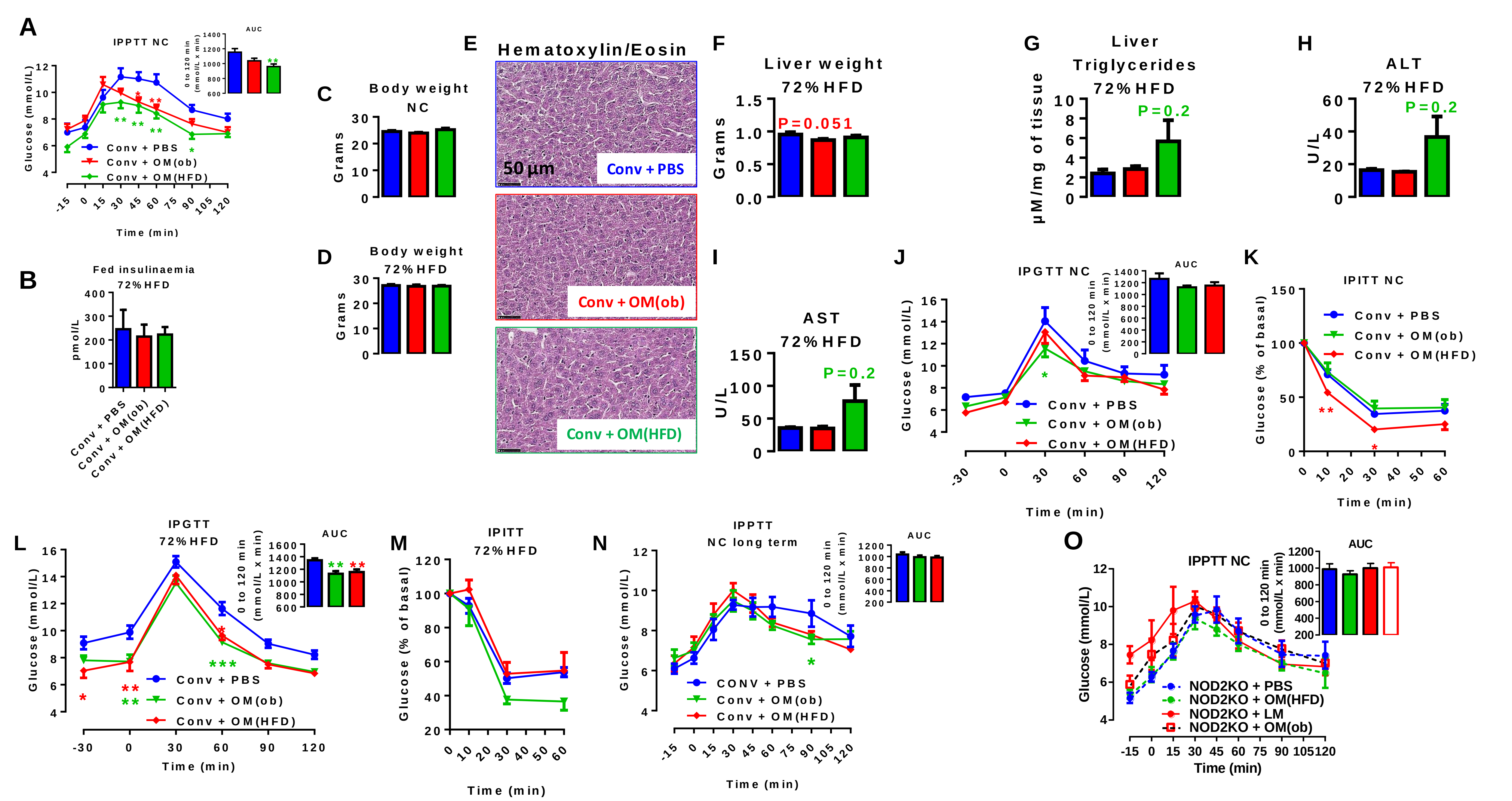

Appendix Figure S6

Appendix Figure S6. Hepatic metabolism during transfer of dysbiotic gut microbiota in NC-fed conventional mice then fed a 72%HFD.

A) Intraperitoneal pyruvate-tolerance test on NC and AUC as inset; after switching on 72%HFD: B) Fed insulinaemia; Body weight on C) NC and D) 72%HFD; E) Hepatic histology section on hematoxylin/eosin staining; liver F) weight and G) triglycerides; plasma levels of H) ALT and I) AST; J) IPGTT and AUC as inset on NC; K) IPITT on NC; L) IPGTT and AUC as inset on 72%HFD; M) IPITT on 72%HFD and N) IPPTT NC long term in antibiotic-free NC-fed conventional mice transferred with either the vehicle or cecal microbiota from C57Bl/6 *ob/ob* or HFD-fed mice (Conv + PBS, Conv + OM(ob), Conv + OM(HFD), respectively) and then fed a 72%HFD or left in NC for a long term. For (O) intraperitoneal pyruvate-tolerance test and AUC as inset in conventional NOD2 KO mice. Data are shown as mean ± SEM; (n=5-6). $^*p<0.05$, $^{**}p<0.01$, $^{***}p<0.001$, unpaired Student's T-test for insets 5A, L). For (O) (n=3-6); 2-ANOVA with Dunnett post-test (A, J, K, L, N, O).

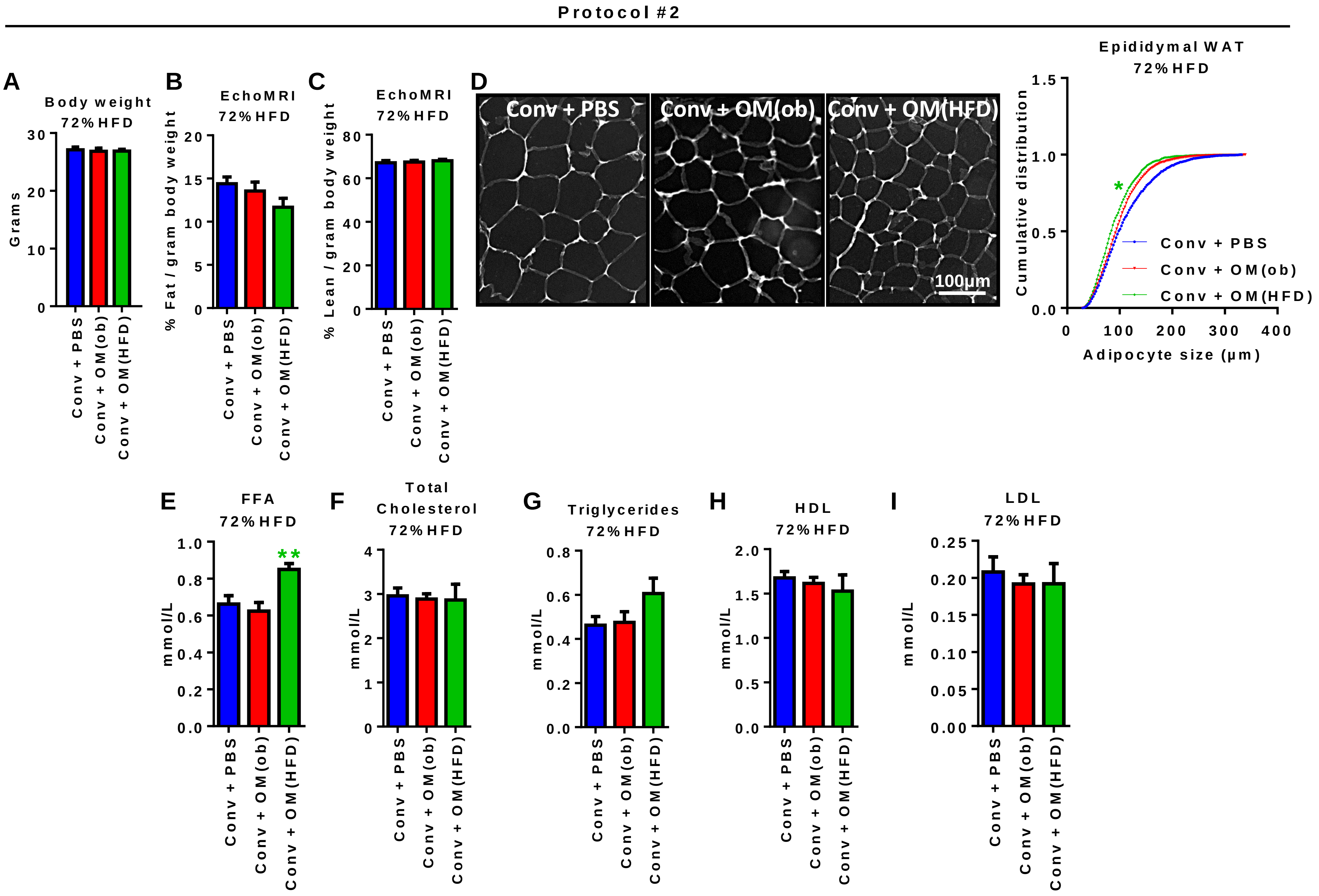

Appendix Figure S7

Appendix Figure S7. Body composition and adiposity during transfer of dysbiotic gut microbiota in NC-fed conventional mice then fed a 72%HFD.

A) Body weight; EchoMRI analysis of B) fat and C) lean mass (%); D) Epididymal WAT histology with cumulative distribution of WAT adipocytes; plasma levels for E) free-fatty acids, F) total cholesterol, G) triglycerides, H) HDL and I) LDL cholesterol in antibiotic-free NC-fed conventional mice inoculated with either vehicle or cecal microbiota from C57Bl/6 *ob/ob* or HFD-fed mice (Conv + PBS, Conv + OM(ob), Conv +OM(HFD), respectively) and then fed a 72%HFD. Data are shown as mean ± SEM; (n=5-6, *$p<0.05$, 2-ANOVA and Sidak's post-test vs. Conv + PBS for D and **$p<0.01$, unpaired Student's T-test vs. Conv + PBS for E).

Protocol #2

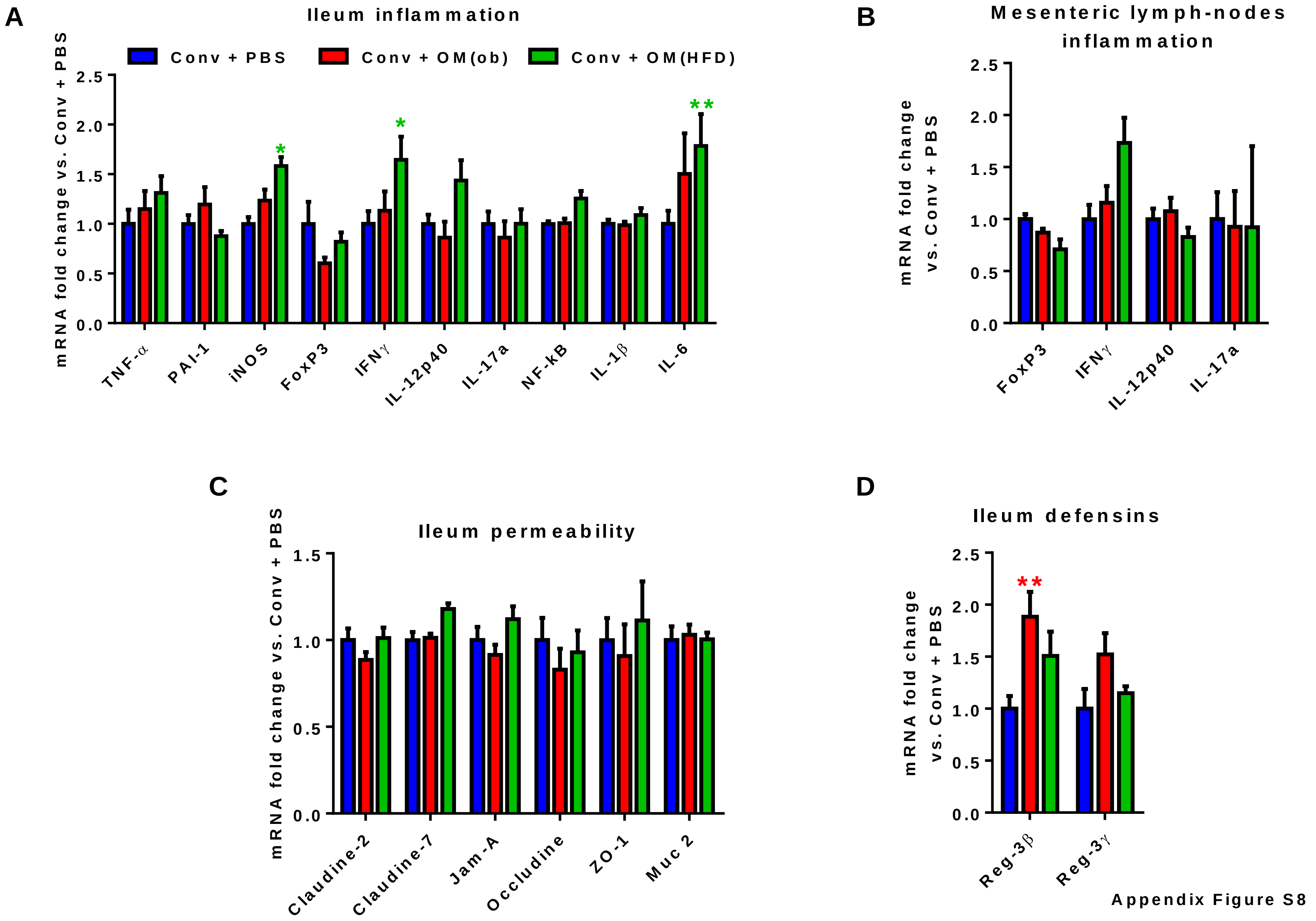

Appendix Figure S8

Appendix Figure S8. Intestinal (ileum) inflammation and permeability during transfer of dysbiotic gut microbiota in antibiotic-free NC-fed conventional mice then fed a 72%HFD.

Gene expression analysis for inflammatory genes in the ileum (A) and mesenteric lymph-node (B), tight-junction proteins (C) and defensins (D) in antibiotic-free NC-fed conventional mice inoculated with either the vehicle or cecal microbiota from *ob/ob* mice or from C57Bl/6 *ob/ob* or HFD-fed mice (Conv + PBS, Conv + OM(ob), Conv + OM(HFD), respectively) and then fed a 72%HFD. Data are shown as mean ± SEM; (n=3(for D only)-6, *$p<0.05$, **$p<0.01$, 2-ANOVA and Dunnett post-test vs. Conv + PBS (A,C)).

A

a: Atopobium
b: Bacteroides
c: Bacteroidaceae
d: Alkalitalea
e: Marinilabiliaceae
f: Barnesiella
g: Tannerella
h: Porphyromonadaceae
i: Alistipes
j: Rikenellaceae
k: Bacteroidales
l: Sphingobacterium
m: Sphingobacteriaceae
n: Sphingobacteriales
o: Clostridium
p: Clostridiaceae
q: Eubacterium
r: Eubacteriaceae
s: Desulfosporosinus
t: Eubacterium_Erysipelotricl
u: Erysipelotrichaceae
v: Erysipelotrichales

B

a: Atopobium
b: Olsenella
c: Coriobacteriaceae
d: Coriobacteriales
e: Bacteroides
f: Bacteroidaceae
g: Alkalitalea
h: Marinilabiliaceae
i: Barnesiella
j: Tannerella
k: Porphyromonadaceae
l: Prevotella
m: Prevotellaceae
n: Rikenella
o: Ruminofilibacter
p: Bacteroidales
q: Flavobacteriaceae
r: Flavobacteriales
s: Sphingobacterium
t: Sphingobacteriaceae
u: Sphingobacteriales
v: Lactobacillus
w: Lactobacillaceae
x: Lactobacillales
y: Allobaculum
z: Eubacterium_Erysipelotricl
a0: Erysipelotrichaceae
a1: Erysipelotrichales
a2: Desulfovibrio
a3: Desulfovibrionaceae
a4: Desulfovibrionales
a5: Desulfuromonas
a6: Desulfuromonadaceae
a7: Desulfuromonadales

C

a: Barnesiella
b: Butyricimonas
c: Parabacteroides
d: Porphyromonas
e: Prevotella
f: Prevotellaceae
g: Rikenella
h: Gelidibacter
i: Pedobacter
j: Sphingobacterium
k: Oxobacter
l: Sporanaerobacter
m: Oribacterium
n: Roseburia
o: Lachnospiraceae
p: Hydrogenoanaerobacteriu
q: Allobaculum
r: Eubacterium_Erysipelotricl
s: Erysipelotrichaceae
t: Erysipelotrichales
u: Parasutterella
v: Sutterellaceae
w: Burkholderiales
x: Desulfovibrio
y: Desulfovibrionaceae
z: Desulfovibrionales
a0: Akkermansia
a1: Verrucomicrobiaceae
a2: Verrucomicrobiales

Appendix Figure S9. Full list for Cladograms reported in Figure 7.
A) Lower panel for Fig.7A; B) Lower panel for Fig.7B; C) Lower panel for Fig.7C

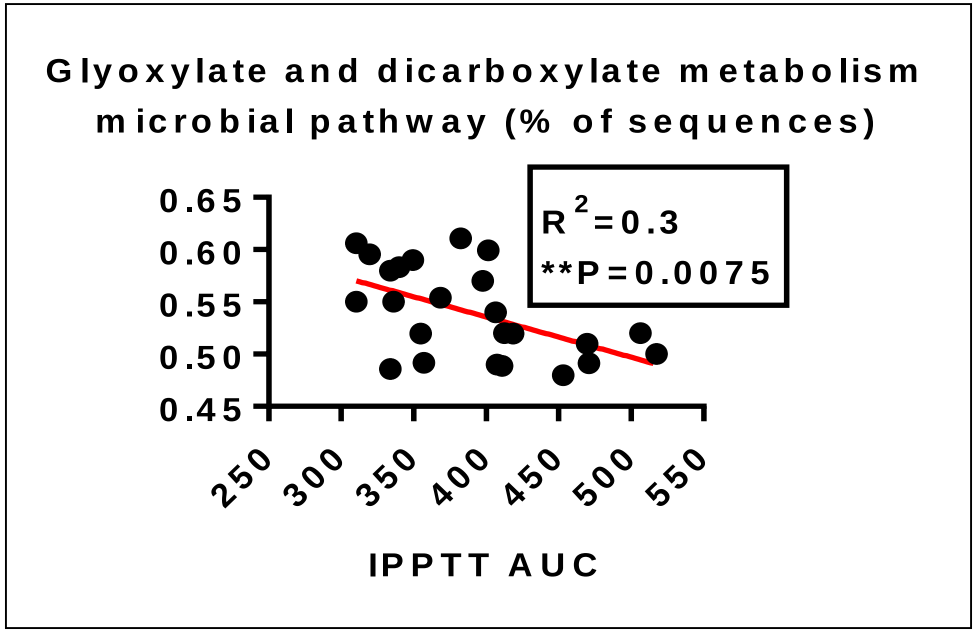

Appendix Figure S10. Glyoxylate and dicarboxylate metabolism microbial pathway correlation with IPPTT AUC.

Protocol #2

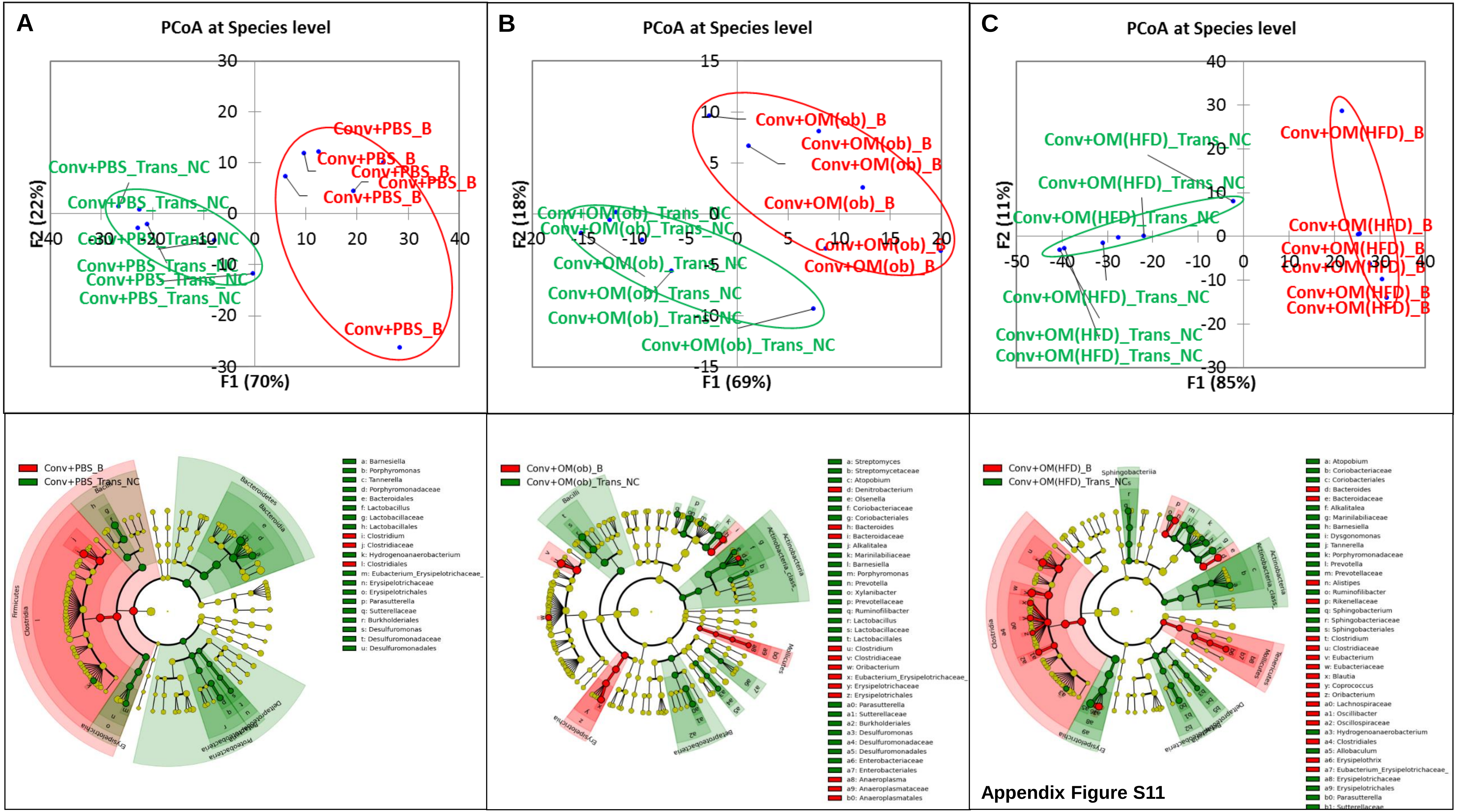

Appendix Figure S11

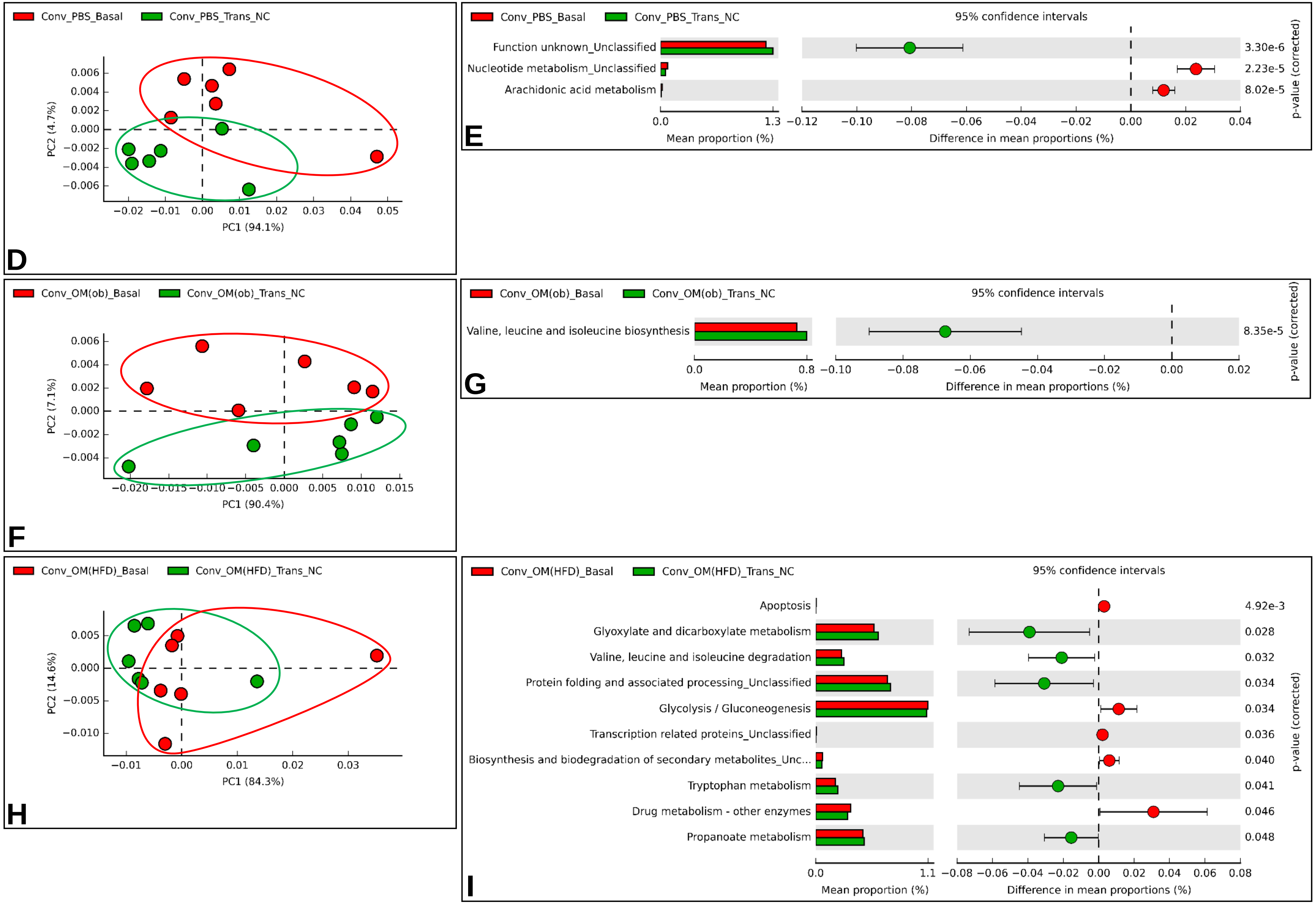

Appendix Figure S11

Appendix Figure S11. Transfer of dysbiotic gut microbiota in antibiotic-free NC-fed conventional mice changes gut microbiota and microbiome according to the origin of dysbiosis.

A) Principal coordinate analysis (PCoA) for gut microbiota profiles at baseline (Basal, B) and after transfer with the vehicle (PBS) on NC (Trans NC) (upper panel) and related cladograms showing bacterial taxa significantly enriched in each group (lower panel); B) PCoA for gut microbiota profiles at baseline (Basal, B) and after transfer with the ob-microbiota (OM(ob)) on NC (Trans NC) (upper panel) and related cladogram (lower panel); C) PCoA for gut microbiota profiles at baseline (Basal, B) and after transfer with the HFD-microbiota (OM(HFD)) on NC (Trans NC) (upper panel) and related cladogram (lower panel); Principal Component Analysis showing PICRUSt-based gut microbiome study at baseline (Basal) and after transfer with the vehicle (PBS, D) or ob-microbiota (OM(ob), F) or HFD-microbiota (OM(HFD), H) on NC (Trans NC) and top modulated (based on the Two sided Welch's t-test) microbial pathways in a pair-wise comparison (E, G, I) in antibiotic-free NC-fed conventional mice inoculated with either the vehicle or cecal microbiota from C57Bl/6 *ob/ob* or HFD-fed mice (Conv + PBS, Conv + OM(ob), Conv + OM(HFD), respectively) and then fed a 72%HFD; (n=5-6).

Protocol #2

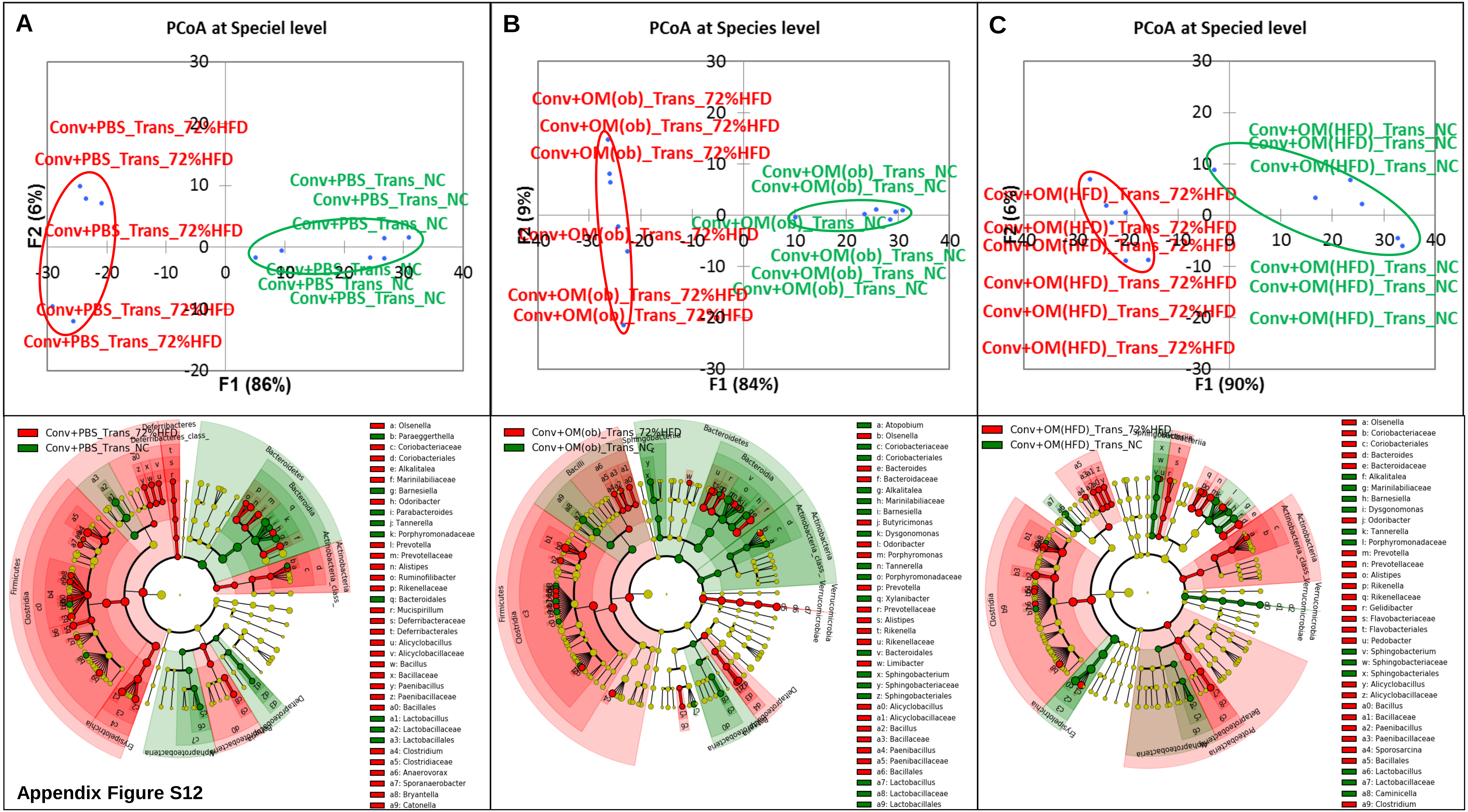

Appendix Figure S12

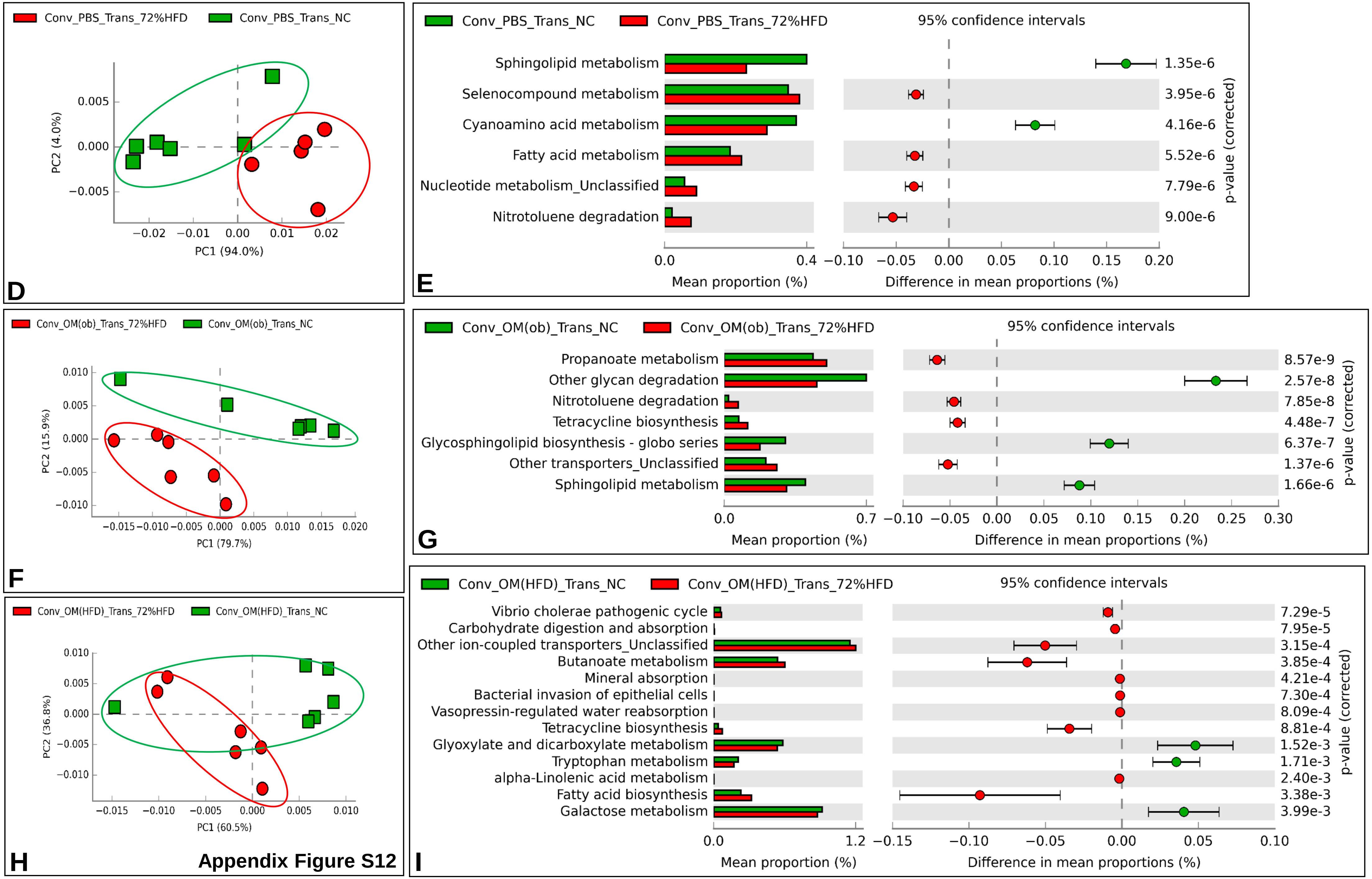

Appendix Figure S12

Appendix Figure S12. Transfer of dysbiotic gut microbiota in antibiotic-free NC-fed conventional mice changes gut microbiota and microbiome according to the origin of dysbiosis. Comparison between NC and 72%HFD nutritional states.

Principal coordinate analysis (PCoA) for gut microbiota profiles between NC (Trans_NC) and 72%HFD (Trans_72%HFD) nutritional states after transfer with A) vehicle (PBS), B) ob-microbiota (OM(ob)), C) HFD-microbiota (OM(HFD)) and related cladogram showing bacterial taxa significantly enriched in each group (lower panel); Principal Component Analysis showing PICRUSt-based gut microbiome study for the groups reported above (D, F, H) and top modulated (based on the Two sided Welch's t-test) microbial pathways in a pair-wise comparison (E, G, I) in antibiotic-free NC-fed conventional mice inoculated with either the vehicle or cecal microbiota from C57Bl/6 *ob/ob* or HFD-fed mice (Conv + PBS, Conv + OM(ob), Conv + OM(HFD), respectively) and then fed a 72%HFD; (n=5-6).
