# Supplementary material for: Transfer of dysbiotic gut microbiota has beneficial effects on host liver metabolism
Source: Mol Syst Biol. 2017 Mar 16;13(3):921. doi: 10.15252/msb.20167356 (PMC5371731; doi:10.15252/msb.20167356)

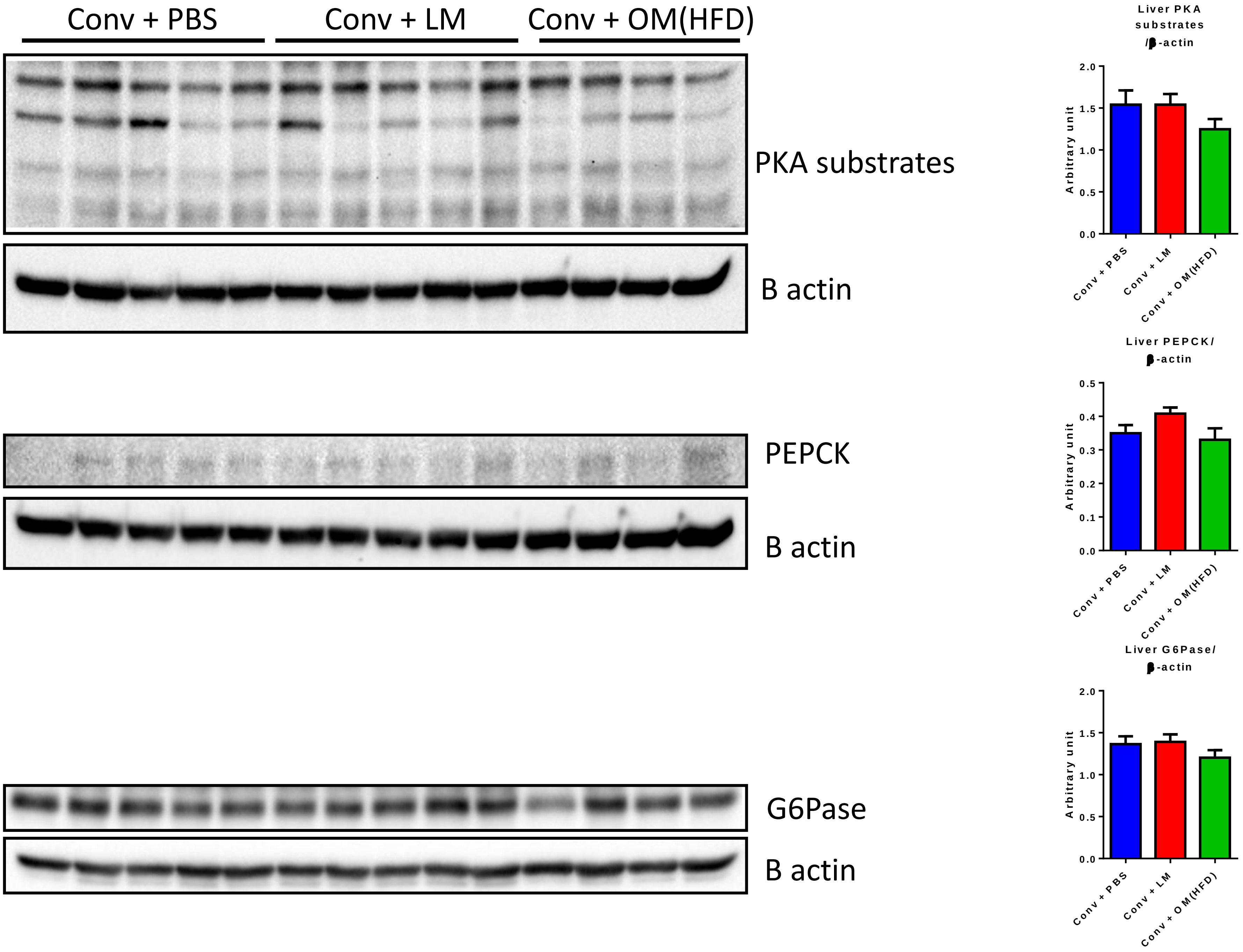
Conv + PBS
Conv + LM
Conv + OM(HFD)
PKA substrates
B actin
PEPCK
B actin
G6Pase
B actin
Liver PKA substrates /β-actin
Liver PEPCK/ β-actin
Liver G6Pase/ β-actin
Arbitrary unit
2.0
1.5
1.0
0.5
0.0
0.5
0.4
0.3
0.2
0.1
0.0
Conv + PBS
Conv + LM
Conv + OM(HFD)

XRS 2015-09-09 10hr 47min-1_Exposure_50.5sec pkas sn4 9915 - Analyse

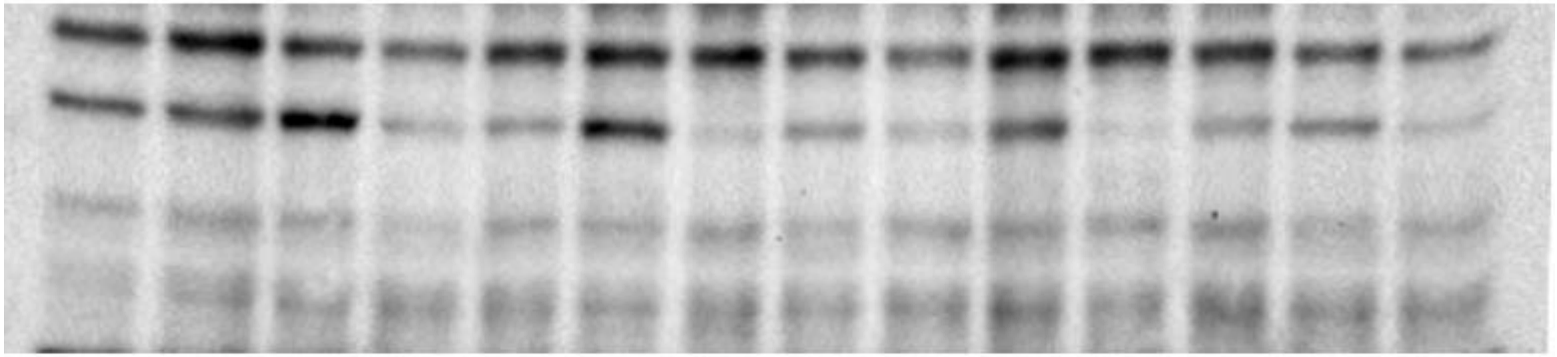

XRS 2015-09-10 11hr 22min_Exposure_4.9sec beta actine pkas 10915 analyse SN4

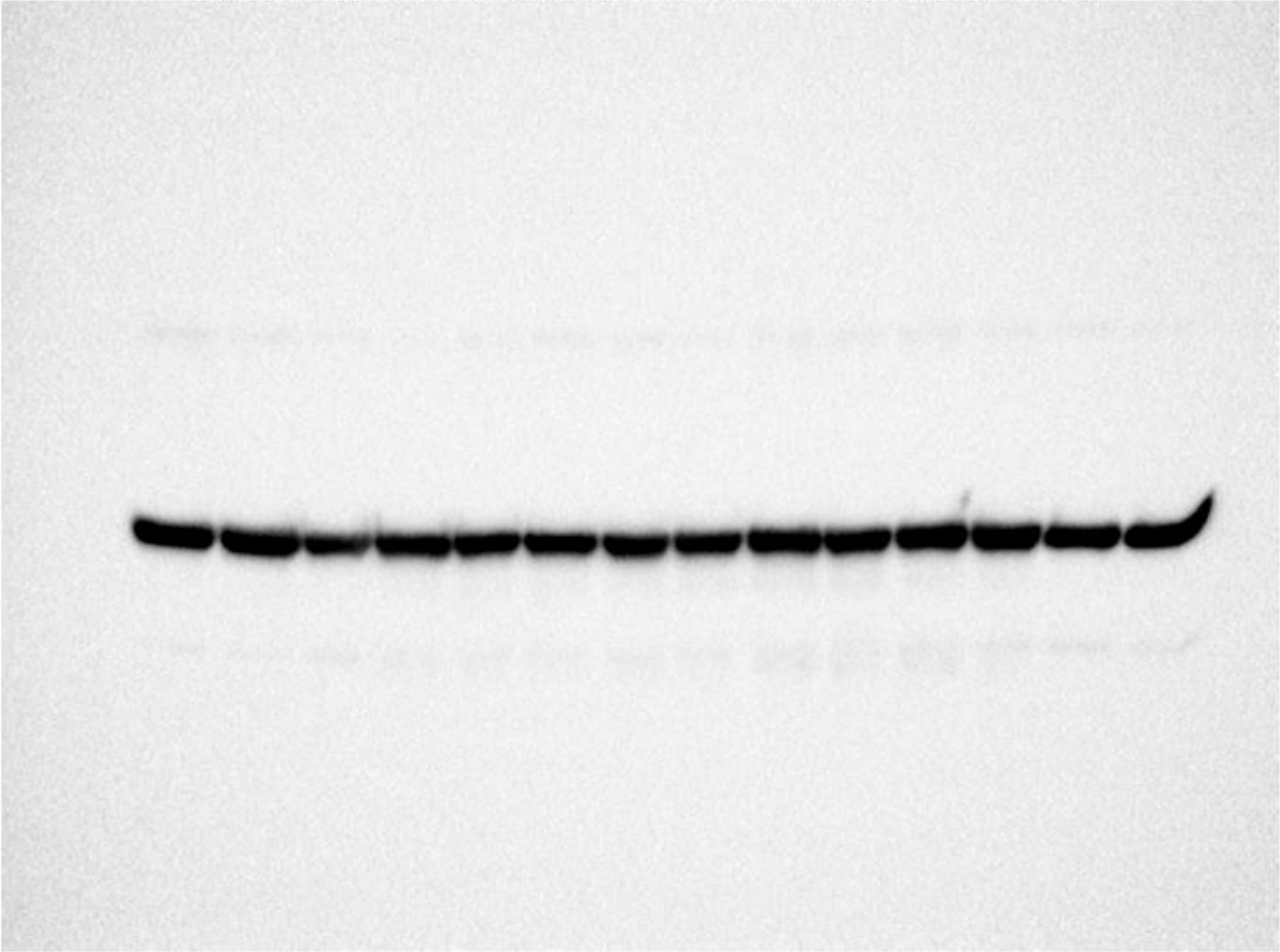

XRS 2015-09-09 10hr 53min_Exposure_120.0sec pepck sn4 9 9 15 Analyse

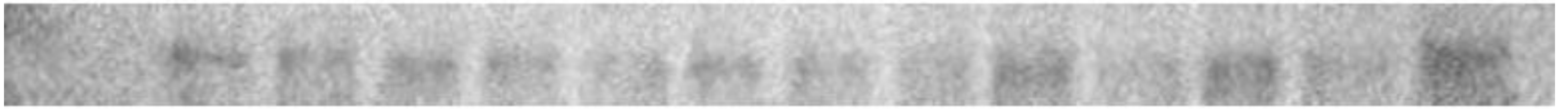

XRS 2015-09-10 11hr 20min_Exposure_4.5 sec beta actine pck1 sn4 10915 - Analyse

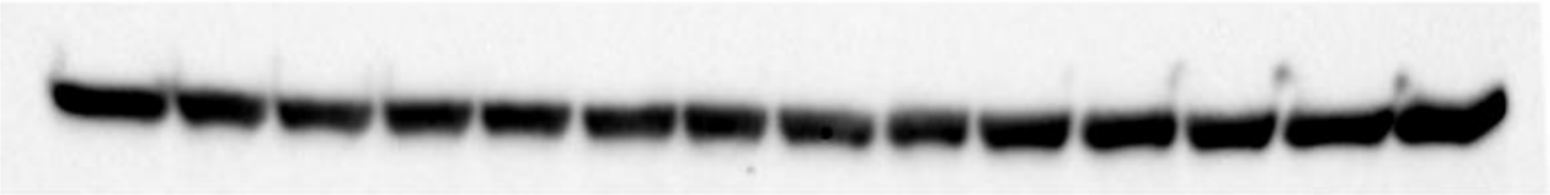

XRS 2015-09-09 10hr 44min-1_Exposure_12.6sec g6p ase 9915 SN4 Analyse

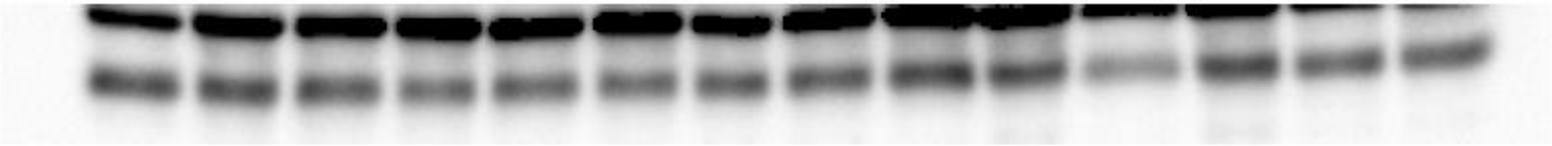

XRS 2015-09-10 11hr 18min_Exposure_7.0sec beta actine g6pase sn4 10915 - Analyse

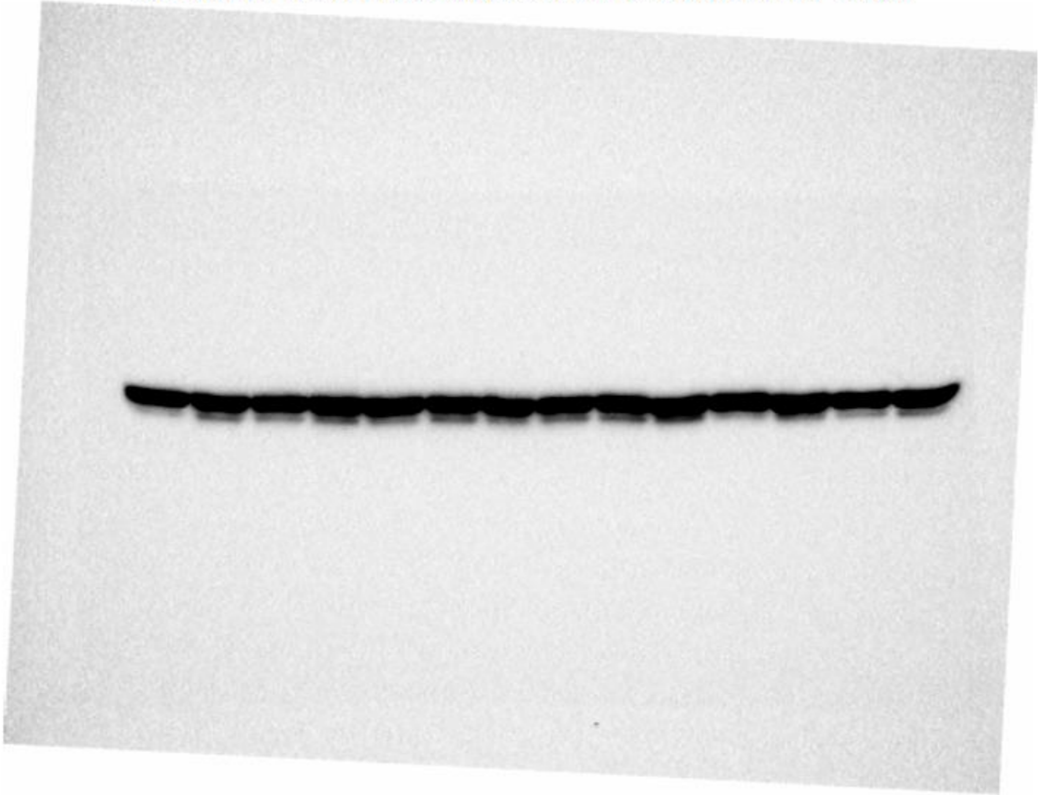

Supplement: Supplementary file 3 — Source Data for Appendix [file MSB-13-921-s004.zip › SourceData_Appendix_FigureS2.pdf]
