# Supplementary material for: Transfer of dysbiotic gut microbiota has beneficial effects on host liver metabolism
Source: Mol Syst Biol. 2017 Mar 16;13(3):921. doi: 10.15252/msb.20167356 (PMC5371731; doi:10.15252/msb.20167356)

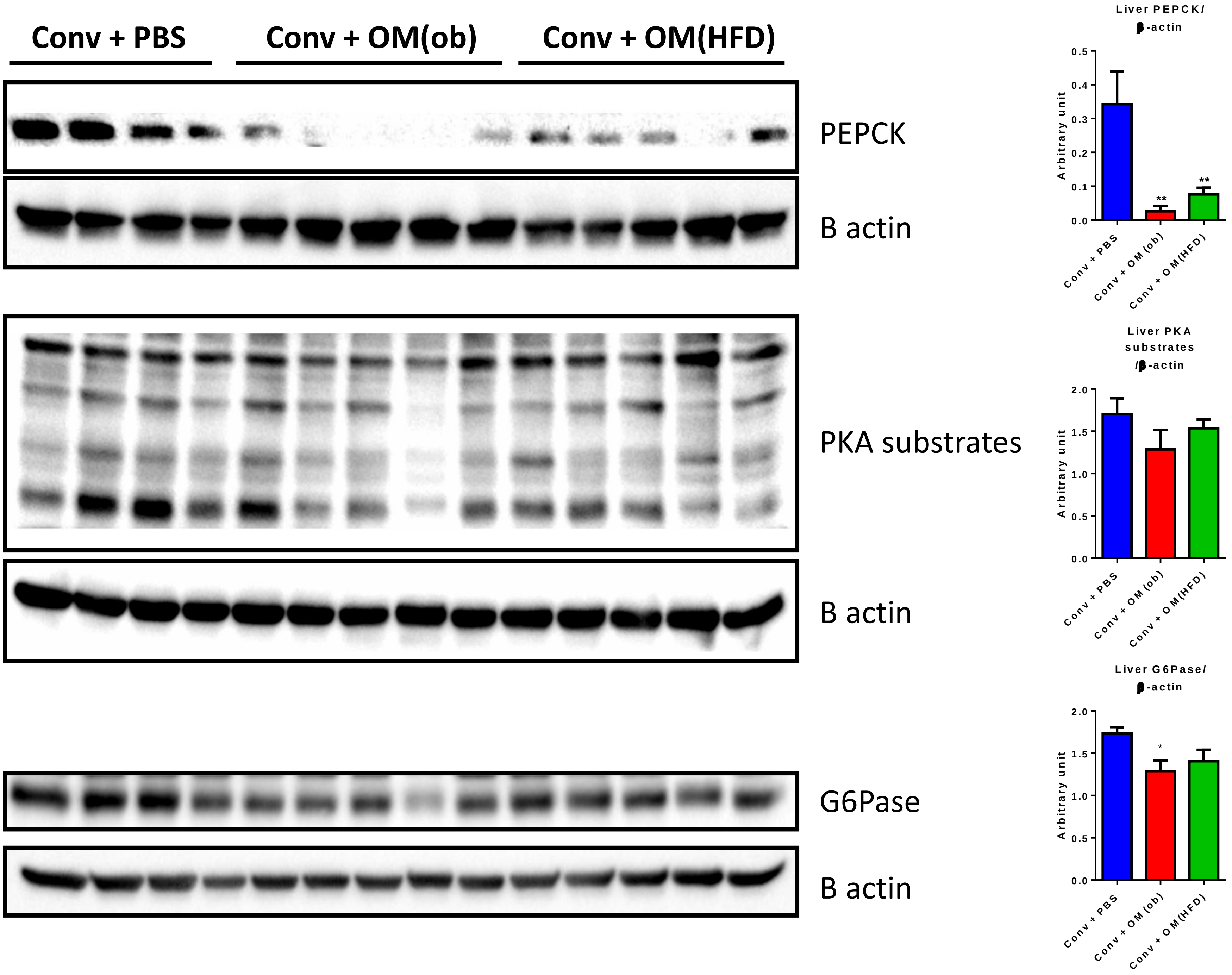
Conv + PBS
Conv + OM(ob)
Conv + OM(HFD)
PEPCK
B actin
PKA substrates
B actin
G6Pase
B actin
Liver PEPCK/ β-actin
Arbitrary unit
0.5
0.4
0.3
0.2
0.1
0.0
**
**
Conv + PBS
Conv + OM (ob)
Conv + OM(HFD)
Liver PKA substrates /β-actin
Arbitrary unit
2.0
1.5
1.0
0.5
0.0
Conv + PBS
Conv + OM (ob)
Conv + OM(HFD)
Liver G6Pase/ β-actin
Arbitrary unit
2.0
1.5
1.0
0.5
0.0
*
Conv + PBS
Conv + OM (ob)
Conv + OM(HFD)

PEPCK SN1 liver photo

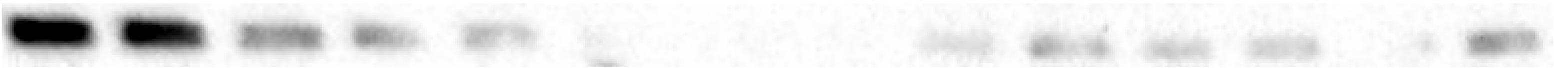

XRS 2015-08-28 10hr 50min beta actine pck1 simon analyse

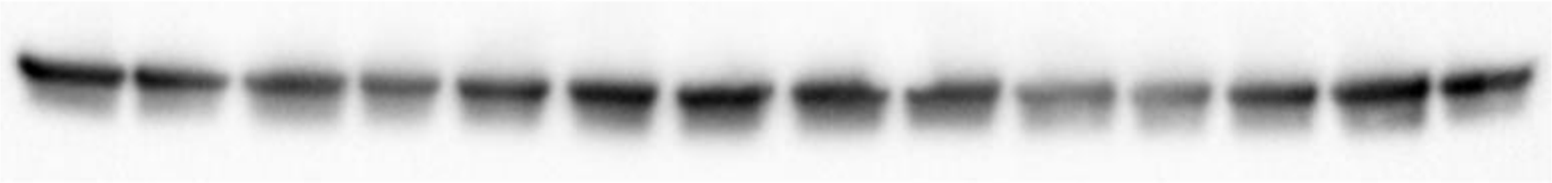

jjm 2015-08-27 10hr 53min_Exposure_35.2sec Photo PKA

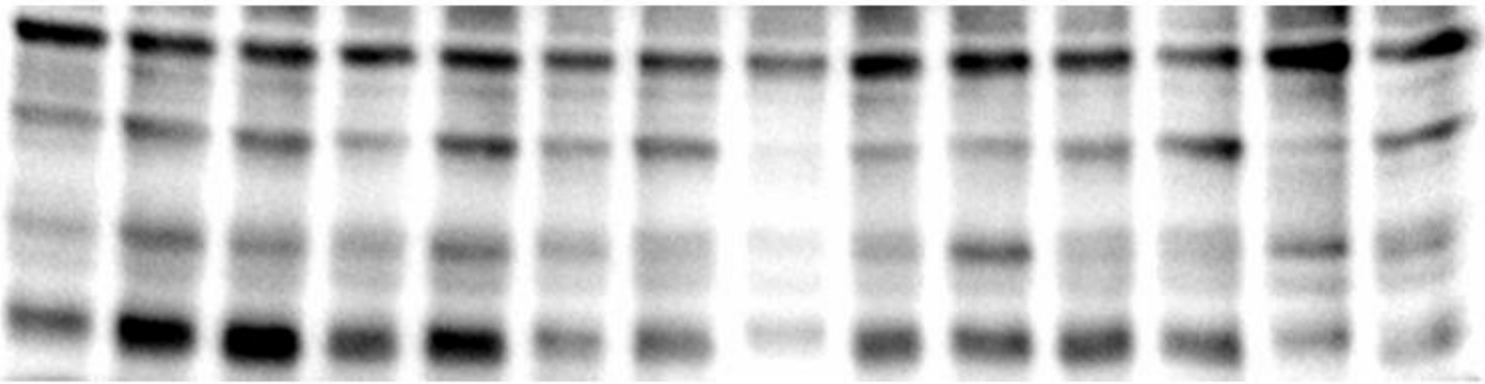

XRS 2015-08-28 beta actine pkas photo

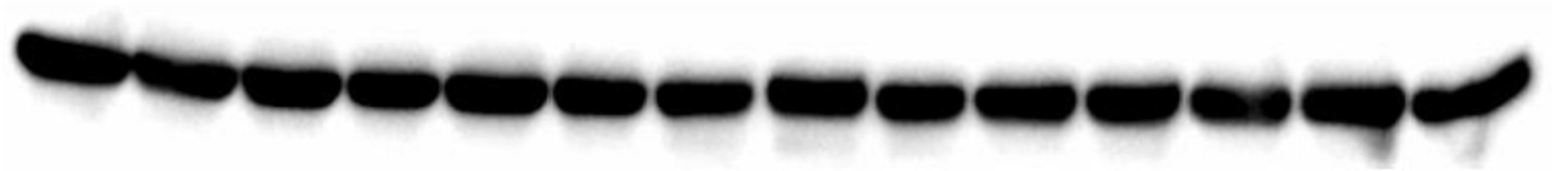

XRS 2015-09-09 10hr 41min_Exposure_14.5sec g6p ase sn1 9915 quantification

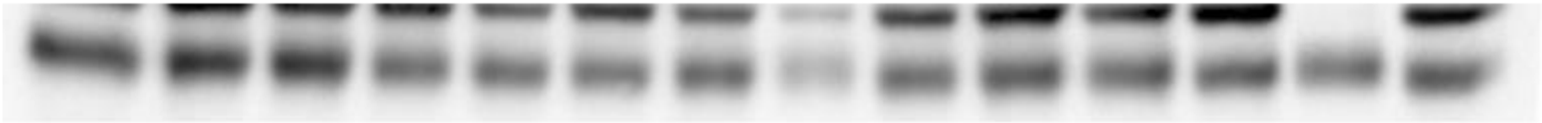

XRS 2015-09-10 11hr 15min_Exposure_7.0secbeta actine sn1 10915 analyse

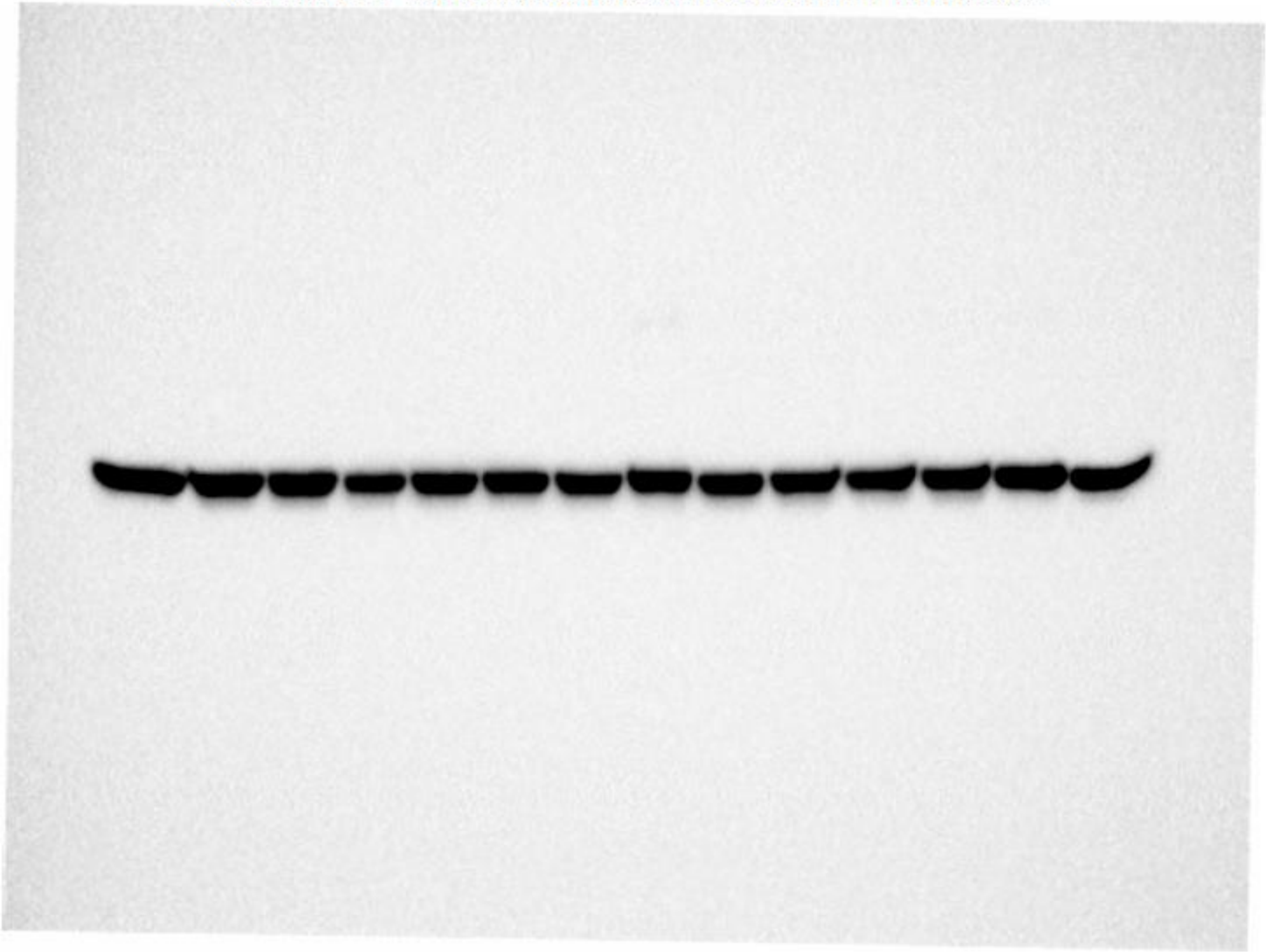

Supplement: Supplementary file 5 — Source Data for Figure 6 [file MSB-13-921-s003.pdf]
